# The challenge of detecting recent natural selection in human populations

**DOI:** 10.1073/pnas.2203237119

**Published:** 2022-03-30

**Authors:** Melinda C. Mills, Iain Mathieson

**Affiliations:** ^a^Leverhulme Centre for Demographic Science, Nuffield College, University of Oxford, Oxford OX1 1JD, United Kingdom;; ^b^Department of Genetics, Perelman School of Medicine, University of Pennsylvania, Philadelphia, PA 19104

A long-term goal of human evolutionary genetics has been to infer and characterize natural selection across broad timescales and geography. In any particular environment, genetic variants that either increase or decrease fitness are more likely to increase or decrease their frequency in the next generation, compared to variants with no effect. These changes in frequency leave distinctive patterns of variation in the genomes of descendant populations and can therefore be inferred indirectly from the genomes of present-day individuals or directly from time series of ancient genomes (1, 2). Typically, these studies are powered to detect selection on timescales of thousands of, tens of thousands of, or more years in the past. In the past few years, however, very large datasets such as the UK Biobank have enabled researchers to look for selection that is extremely recent—on the timescale of decades—or even selection that is ongoing. Despite the difficulties of interpreting these results (3), such studies can potentially provide enormous insight into human evolution, demography, and the relationship between contemporary environment and genotype.

## Environment Drives Genotype Frequencies

Along these lines, in PNAS, Wu et al. (4) describe an approach to identify genetic variants associated with increased fitness. The technique that they use is a geographically based “regional genome-wide association study” (GWAS) (5) that tests for association between an individual’s genotype and the infant mortality rate (IMR) for the time and place of that individual’s birth. It is worth thinking about why this approach, which inverts the standard GWAS interpretation, should work. In a standard GWAS, generally conducted in a large sample of unrelated individuals, inherited genetic variants are associated (i.e., correlated) with a particular phenotype (for example, a disease). Since genotype is assigned at conception and cannot be modified by phenotype, in principle these associations represent causal effects of genotype on phenotype and they are typically interpreted as such. In contrast, since Wu et al. are testing for the association between individual genotype and environment, the interpretation of causality is in the other direction; environment drives genotype frequencies. Specifically, Wu et al. argue that genetic variants that are more common than expected in “poor” environments (represented by high IMR in the year of birth) are common because they provide some fitness advantage in those environments; that is, they are under natural selection. Wu et al. identify two loci—*LCT* and *TLR1/6/10*—as having experienced selection. These loci, which are associated with adult lactase persistence and immune function, respectively, are known targets of selection in the past 8,000 y (6). Indeed, *LCT* exhibits the strongest known signal of selection in the entire human genome. The authors also find significant genetic correlations with polygenic traits that may have also been associated with fitness.

## A Challenging Study Design

The Achilles’ heel of the standard GWAS approach is population stratification. All human populations, including the UK Biobank cohort (5, 7), show some degree of structure. The most obvious example is geographic structure—due to nonrandom mating based on geography, some genetic variants are more common in some parts of the country than others. If the phenotype being tested also varies geographically, then some of those variants may be associated with it, simply by chance. Other types of nonrandom mating, for example, based on social structure or phenotype, have a similar effect. Similarly, the frequency of genetic variants may change over time due to genetic drift, demographic processes, or sampling biases. Such variants would then be associated with phenotypes that change over time for any reason. Very large GWAS may exacerbate the problem by providing statistical power to pick up even very modest stratification due to recent demographic history (8). Of course, this problem is well known, and correction for population structure is a standard part of any GWAS pipeline. Common approaches include the use of genomic control (GC) (9), inclusion of principal components (PCs) of genome-wide data as covariates in the linear regression model (10), or the use of a mixed model where one of the error terms has a covariance structure equal to the kinship matrix of the individuals in the study (11). However, none of these methods is perfect. Analyses that combine information across many variants, for example, polygenic scores, genetic correlations, and tests for polygenic selection, are particularly sensitive to residual population stratification in even relatively well-controlled GWAS (7, 12, 13). It is important to realize that no correction can be guaranteed to remove the effects of stratification completely; rather, we can only hope that it has been reduced to an acceptable level.

The study design of Wu et al. (4) is vulnerable to the same issue. Since IMR varies dramatically across space and time, genetic variants that are geographically or temporally structured may be associated with it despite there being no causal relationship. The authors are aware of this and go to great lengths to correct for stratification. They apply GC, PCs, and mixed model corrections, as well as testing robustness of their results to inclusion of linear effects of educational attainment and household income. It is difficult to see what more could be done in a GWAS context. Yet, at the same time, it is impossible to be certain that all stratification has been removed. The two genome-wide significant loci in the study are two of the most significantly geographically structured loci across regions of Britain in the entire genome (14). Similarly, educational attainment, which is the trait with the largest genetic correlation with IMR, is highly stratified in the UK Biobank even after adjustment for 40 principal components (7). These observations immediately raise the prospect of uncontrolled stratification, despite the authors’ best efforts. Of course, one could make the counterargument that this is exactly what one would expect. These loci are geographically structured precisely because of historical selection. We know, therefore, that they can affect fitness under certain conditions and so it should come as no surprise to find that they affect fitness in more recent times. Whether we think that geographic structure is leading to spurious signals of selection or that recent selection is creating geographic structure comes down to whether we believe the controls for stratification in the GWAS are good enough. But this is difficult or impossible to know.

Other effects also complicate the interpretation of these results. One is assortative mating, which refers to the fact that individuals do not choose partners at random but rather often based on proximity along environmental strata patterned by geography, education, income, religion, ethnicity and family circumstances (15). This induces stratification directly related to environment. Second is the issue of nonrandom participation in the UK Biobank, with a recent GWAS demonstrating that, beyond mortality selection and survival, the inclination to participate in the UK Biobank is associated with educational attainment, body mass index (BMI), and participation in a dietary study (16). Finally, GWAS results may also contain traces of indirect genetic effects of the parents through untransmitted alleles of genetic nurture, or, in other words, although the parents do not transmit all of their alleles during meiosis, the environment they produce still contains that genetic nurture (17). Wu et al. (4) do acknowledge and address some of these concerns, but, as with stratification, the GWAS study design is fundamentally vulnerable to these effects.

## Alternative Approaches

How might we overcome these vulnerabilities? Replicating these results in other populations, even in other northern European countries, would go a long way toward demonstrating that they are not an artifact of population structure in Britain. Another way forward is to collect large-scale family-based data and conduct family-based association tests that can properly control for both genetic nurture and demography. In addition to the technique the authors (4) use in this study to parse out direct and indirect genetic effects using multigenerational GWAS (18), sibling-based studies are another way forward. These reveal differences in trait outcomes by comparing biological full siblings, regressed on differences in their genotype. A recent large-scale study of up to ∼160,000 siblings showed that GWAS associations overestimated direct effects across a wide array of phenotypes, particularly nonclinical behavioral ones (19). We know, from a growing number of studies, that, when within-sibship effect size estimates are used, some genetic correlations, such as between educational attainment and height (19) or education and BMI (20, 21), largely disappear.

In PNAS, Wu et al. describe an approach to identify genetic variants associated with increased fitness. The technique that they use is a geographically based “regional genome- wide association study” (GWAS) that tests for association between an individual’s genotype and the infant mortality rate (IMR) for the time and place of that individual’s birth.

More generally, confidence in these statistical associations would be increased if they could be placed in a broader evolutionary context. For example, *LCT* exhibits one of the strongest signals of historical natural selection in the entire genome. Even so, the actual basis of this selective pressure remains unclear (22, 23), although the prevalent explanation is that it is ultimately driven by selection for increased calcium levels. Direct data from ancient DNA show that the frequency of the persistence allele in Britain increased rapidly from about 4,000 y to 2,000 y before present, but has not changed substantially for the last 2,000 y (24), indicating a lack of consistent selective pressure for most of that time. Could such a pressure nonetheless have existed for a few years in the middle of the 20th century? Perhaps, and, if so, understanding why might contribute greatly to our understanding of historical selective pressures. However, given the statistical limitations of the approach, the lack of a convincing biological basis or evolutionary explanation for the observations raises doubts. Fortunately, studying the environmental drivers of near-contemporary selection is much more achievable than for ancient selection, so the question may not be completely intractable.

## The Limits of GWAS

So, does the Wu et al. (4) study provide evidence of recent natural selection? Does it, as the authors claim, “directly estimate the shift of allele frequencies in less favorable environments”? The study design is innovative and perhaps at the limits of what one could achieve in this area with GWAS-based design alone. However, we find ourselves cautiously unconvinced. The results from these types of designs alone might be positioned more modestly as hypothesis generation rather than definitive evidence. This approach is an exciting first step, but it needs to be confirmed with independent approaches that do not suffer from the same statistical limitations, across other contexts and in cohorts outside of the United Kingdom. The results also need to be situated in a broader biological and evolutionary context. This study therefore illustrates the potential of large genomic datasets to detect and quantify recent natural selection but also highlights the fundamental limitations and difficulty of interpretation of the GWAS study design.
